# Early Diagnosis and Management of Arthrogryposis Multiplex Congenita in a Neonate: A Case Study

**DOI:** 10.7759/cureus.60729

**Published:** 2024-05-21

**Authors:** Astha Khurana, Amar Taksande, Revat J Meshram, SreeHarsha Damam, Chaitanya Kumar Javvaji, Kushal Desai, Ankita Patel, Rahul Khandelwal

**Affiliations:** 1 Pediatrics, Jawaharlal Nehru Medical College, Datta Meghe Institute of Higher Education and Research, Wardha, IND

**Keywords:** multidisciplinary care, prenatal ultrasound, respiratory distress, joint contractures, neonate, arthrogryposis multiplex congenita

## Abstract

Arthrogryposis multiplex congenita (AMC) is a rare condition characterized by multiple joint contractures at birth, affecting two or more body areas. The clinical examination revealed physical abnormalities indicative of AMC, including joint contractures, clubfeet, and scoliosis. The diagnostic evaluation confirmed the clinical suspicion, and prompt management was initiated to address respiratory distress and potential sepsis. Early diagnosis and multidisciplinary care are essential for optimizing outcomes in neonates with AMC. We present the case of a one-day-old neonate who exhibited immediate respiratory distress upon birth and was born via a lower segment cesarean section (LSCS) to a 31-year-old mother. This case underscores the importance of recognizing prenatal ultrasound findings suggestive of AMC and implementing appropriate postnatal care strategies for affected neonates. Early diagnosis and multidisciplinary care are essential for optimizing outcomes in neonates with AMC.

## Introduction

Arthrogryposis multiplex congenita (AMC) is a rare musculoskeletal disorder characterized by multiple joint contractures present at birth, affecting two or more body areas [1]. The condition encompasses a spectrum of phenotypes ranging from isolated joint contractures to involvement of multiple joints, along with other associated anomalies such as clubfeet, scoliosis, and respiratory difficulties [2]. The etiology of AMC is multifactorial, involving genetic and environmental factors [3]. Genetic factors may include mutations in genes involved in muscle development and function, such as myosin heavy chain 3 (MYH3), tropomyosin 2 (TPM2), and troponin T3 (TNNI2) [4]. Additionally, prenatal environmental factors, such as intrauterine constraint and vascular compromise, have been implicated in the pathogenesis of AMC [5].

Prenatal ultrasound plays a crucial role in the antenatal diagnosis of AMC by detecting abnormalities in fetal limb movements, joint positions, and contractures [6]. Early detection allows for appropriate counseling of parents and facilitates multidisciplinary planning for postnatal care, including delivery considerations and management of associated complications. The management of neonates with AMC requires an interdisciplinary approach involving neonatologists, orthopedic surgeons, geneticists, and physical therapists [7]. Treatment strategies aim to address joint contractures, optimize functional outcomes, and manage associated complications such as respiratory distress and feeding difficulties. Despite advances in prenatal imaging and postnatal care, the prognosis for individuals with AMC varies depending on the severity of joint involvement and associated anomalies. Early recognition, comprehensive evaluation, and prompt initiation of interventions remain paramount to improving outcomes for affected neonates.

## Case presentation

**Figure 1:**
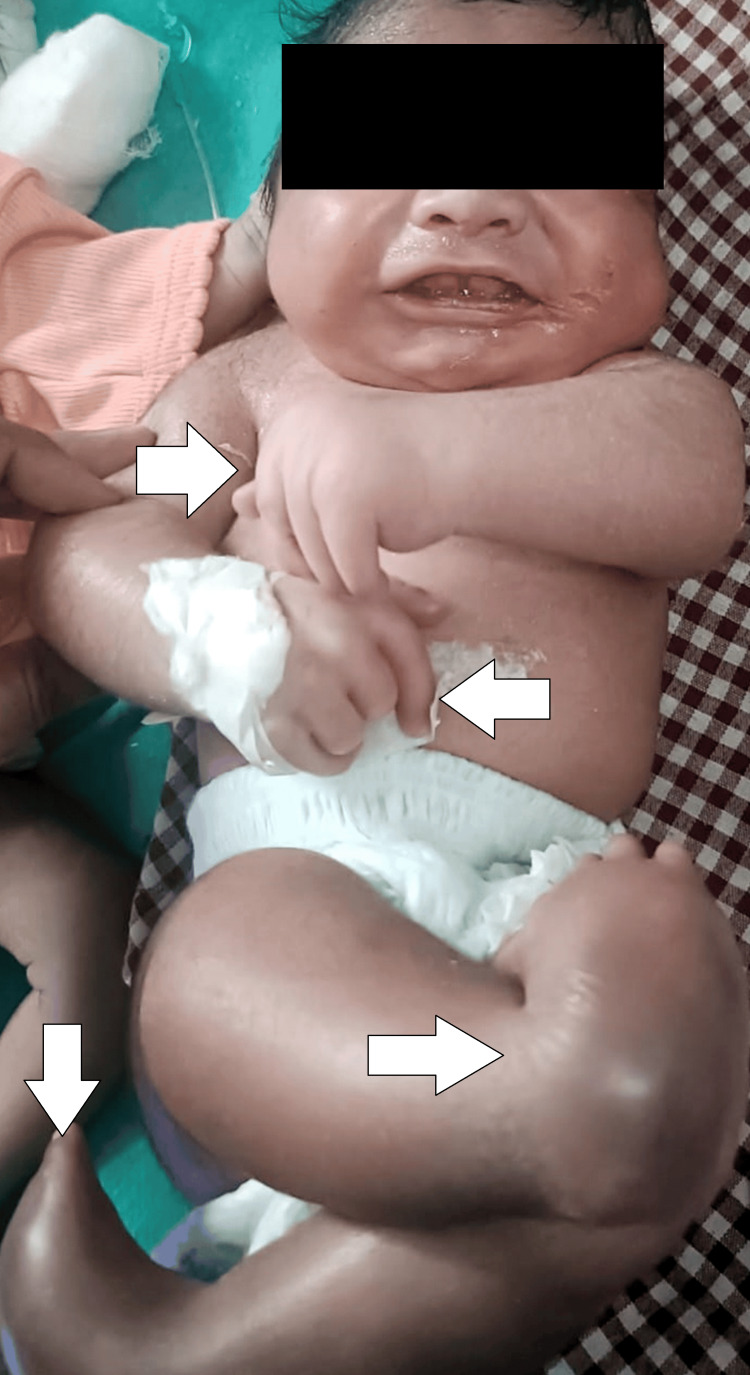
Image of the neonate showing shoulders in adduction and internal rotation, elbows in fixed flexion, wrists in flexion, rigid interphalangeal joints with camptodactyly, partial subluxation of the left hip joint, knees in fixed extension, rigid bilateral clubfeet with vertical talus, and scoliosis (arrows)

We present the case of a one-day-old neonate, born at 38 weeks of gestation, delivered via lower segment cesarean section (LSCS) to a 31-year-old mother, gravida 3, para 3. The mother, in a non-consanguineous marriage, had one previous abortion. The male neonate was the couple's second child, with one living child from a previous pregnancy. Upon birth, the neonate exhibited an immediate cry but quickly developed respiratory distress. Clinical examination revealed a constellation of physical abnormalities indicative of AMC. The neonate displayed shoulders in adduction and internal rotation, elbows in fixed flexion, wrists in flexion, rigid interphalangeal joints with camptodactyly, partial subluxation of the left hip joint, knees in fixed extension, rigid bilateral clubfeet with vertical talus, and scoliosis (Figure 1).

**Figure 2:**
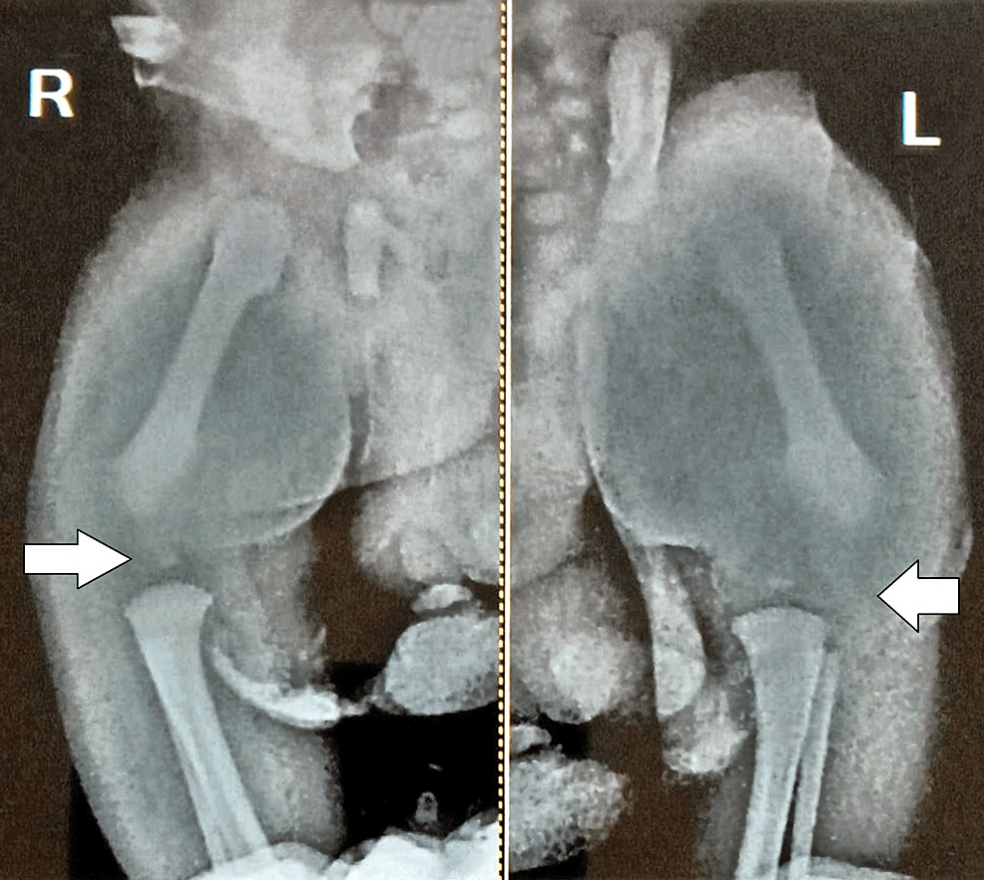
The X-ray of the pelvis revealed left hip joint subluxation (arrows)

These findings raised strong suspicion for AMC. The diagnostic evaluation included X-rays of the pelvis and both hands and wrists, which confirmed the clinical suspicion. The X-ray of the pelvis revealed left hip joint subluxation (Figure 2).

**Figure 3:**
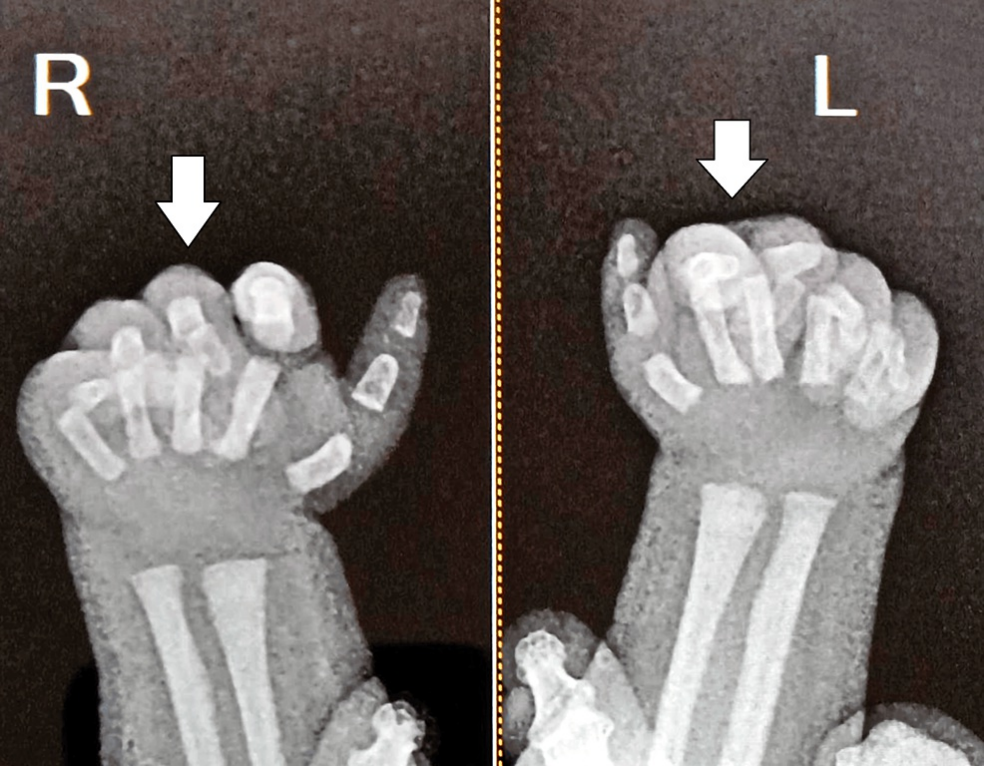
The X-ray of both hands and wrists showed features consistent with AMC, including camptodactyly and radial deviation of the fingers (arrows)

At the same time, the X-ray of both hands and wrists showed features consistent with AMC, including camptodactyly and radial deviation of the fingers (Figure 3). Additionally, the antenatal ultrasound findings had previously indicated abnormal limb/extremity positioning, clubfeet, and scoliosis, further supporting the diagnosis of AMC.

The management of the neonate involved prompt initiation of oxygen support to address respiratory distress. Broad-spectrum antibiotics were administered to cover potential sepsis, given the vulnerability of the neonate and the risk of infection. Nebulization therapy was also commenced to alleviate respiratory symptoms and support lung function. AMC is a rare condition characterized by multiple joint contractures at birth, affecting two or more body areas. The etiology is multifactorial, with genetic and environmental factors playing a role. Prenatal ultrasound findings often raise suspicion for AMC, prompting further evaluation and planning for appropriate postnatal care.

## Discussion

AMC presents a challenging clinical scenario due to its heterogeneous etiology and variable phenotypic expression. In our case, the neonate exhibited classical features of AMC, including joint contractures, clubfeet, and scoliosis, which prompted diagnostic evaluation and the initiation of management strategies. This discussion aims to elucidate the diagnostic approach, management considerations, and potential implications for the prognosis of neonates with AMC. The diagnostic evaluation of AMC involves a thorough clinical examination supplemented by radiographic imaging and, in some cases, genetic testing. Clinical examination typically reveals multiple joint contractures, which may involve the shoulders, elbows, wrists, knees, and ankles, as well as other associated musculoskeletal abnormalities [8]. In our case, the X-rays of the pelvis and extremities confirmed the clinical suspicion, highlighting the utility of radiographic imaging in confirming the diagnosis and assessing the extent of joint involvement [9].

Prenatal ultrasound findings can also raise suspicion for AMC, prompting further evaluation and planning for appropriate postnatal care. Antenatal ultrasound may reveal abnormalities such as limb/extremity positioning, clubfeet, and scoliosis, which are consistent with the diagnosis of AMC [10]. In our case, the antenatal ultrasound findings supported the clinical diagnosis of AMC, underscoring the importance of prenatal screening in identifying fetuses at risk for this condition. The management of neonates with AMC requires a multidisciplinary approach involving specialists in neonatology, pediatric orthopedics, physical therapy, and other relevant disciplines. In our case, the prompt initiation of oxygen support addressed the respiratory distress. At the same time, broad-spectrum antibiotics were administered to cover potential sepsis, given the vulnerability of the neonate and the risk of infection. Nebulization therapy was also initiated to alleviate respiratory symptoms and support lung function. Additionally, early referral to a pediatric orthopedic specialist is crucial for the assessment and management of joint contractures, clubfeet, and other musculoskeletal abnormalities [11]. The prognosis of neonates with AMC varies depending on the severity of joint involvement, associated complications, and response to treatment. Early recognition and intervention can improve outcomes by minimizing joint deformities and optimizing functional abilities. However, AMC may be associated with long-term disability and functional impairment, necessitating ongoing multidisciplinary care and support [9].

## Conclusions

In conclusion, the presented case underscores the critical importance of recognizing prenatal ultrasound findings suggestive of AMC and implementing timely postnatal management strategies. AMC, characterized by multiple joint contractures at birth, poses challenges in diagnosis and management due to its heterogeneous etiology and variable clinical presentation. Early diagnosis facilitated prompt initiation of interventions to address respiratory distress and potential sepsis, highlighting the significance of multidisciplinary care involving neonatologists, pediatric orthopedic specialists, and other healthcare professionals. While AMC may be associated with long-term disability and functional impairment, early recognition and intervention can improve outcomes by minimizing joint deformities and optimizing functional abilities. Further research is warranted to elucidate the underlying genetic and environmental factors contributing to AMC and to develop targeted interventions to improve the long-term prognosis of affected individuals.
